# Potential efficacy of weekly low-dose administration of bevacizumab as a combination therapy for platinum-resistant ovarian carcinoma: a retrospective analysis

**DOI:** 10.1186/s12885-022-09271-3

**Published:** 2022-02-16

**Authors:** Jin Suminokura, Morikazu Miyamoto, Tomoyuki Yoshikawa, Hiroko Kouta, Yoshihiro Kikuchi, Taira Hada, Hiroki Ishibashi, Tsubasa Ito, Hideki Iwahashi, Soichiro Kakimoto, Rie Suzuki, Hiroko Matsuura, Naohisa Kishimoto, Masashi Takano

**Affiliations:** 1 Department of Obstetrics and Gynecology, National Defense Medical College Hospital, 3-2, Namiki, Tokorozawa, Saitama 359-8513 Japan; 2 Department of Clinical Oncology, National Defense Medical College Hospital, 3-2, Namiki, Tokorozawa, Saitama 359-8513 Japan; 3 Department of Gynecology, Ohki Memorial Kikuchi Cancer Clinic for Women, 111-1, Arahata, Tokorozawa, Saitama 359-1133 Japan

**Keywords:** Ovarian carcinoma, Bevacizumab, Administration, Prognosis, Adverse effect

## Abstract

**Background:**

Bevacizumab (Bev) plays the central role of the adjuvant therapy for patients with ovarian carcinoma. The aim of our study was to examine whether differences in the administration of Bev influence the prognosis of patients.

**Methods:**

Patients with ovarian carcinoma who received treatment at two hospitals between 1999 and 2020 were identified. Patients treated with weekly low-dose administration of Bev (100 mg Bev on days 1 and 8 and 200 mg Bev on day 15, monthly) at one hospital (group A) and those with monthly high-dose administration of Bev (15 mg/kg of Bev on day 1, monthly) at another hospital (group B) were retrospectively compared.

**Results:**

Among the total patients, 44 were assigned to group A and 33 were assigned to group B. More patients in group A had advanced disease (*p* = 0.03) and a lower dose of Bev at the first time during the first cycle administration (*p* < 0.01) than in group B. Progression-free survival (PFS) was better in group A than in group B (*p* < 0.01). Multivariate analysis revealed that group A was a better prognostic factor for PFS (hazard ratio 0.53, *p* = 0.03). Stable duration was longer in group A than in group B (*p* < 0.01). The incidences of adverse effects, including hematological toxicities such as neutropenia (*p* = 0.01) and nonhematological toxicities such as hypertension (*p* < 0.01), intestinal obstruction (*p* < 0.01), and thromboembolic events (*p* < 0.01), were lower in group A than in group B.

**Conclusions:**

Weekly low-dose administration of Bev might improve prognosis and decrease the frequency of adverse effects associated with this drug although the prospective study was needed to get corroboration.

## Background

Ovarian carcinoma is the most well-known gynecological cancer worldwide [1]. The standard treatment is the combination of debulking surgery and chemotherapy. Despite advances in treatment, approximately 75% of women with advanced disease experience recurrence, with worse prognosis [2].

Bevacizumab (Bev) is a humanized vascular endothelial factor (VEGF) monoclonal antibody that inhibits tumor angiogenesis [3]. The Gynecologic Oncology Group (GOG)-218 and International Collaboration on Ovarian Neoplasms (ICON)-7 trials demonstrated that Bev as the first-line therapy improved progression-free survival (PFS) in patients with ovarian carcinoma [4, 5]. In addition, the The Ovarian Cancer Study Comparing Efficacy and Safety of Chemotherapy and Anti-angiogenic Therapy in Platinum-Sensitive Recurrent Disease (OCEANS) and Avastin Use in Platinum-Resistant Epithelial Ovarian Cancer (AURELIA) trials showed that Bev improved PFS in patients with platinum-sensitive and platinum-resistant recurrent ovarian carcinoma [6, 7]. However, the main analysis of these studies showed that Bev could not improve overall survival (OS), although the subanalysis demonstrated that only the combination of Bev and paclitaxel could improve OS [8]. Recently, the Randomized, Double-Blind Controll Trial of Olaparib vs. Placebo in Patients with Advanced Ovarian Cancer (PAOLA)-1 trial indicated that Bev added to olaparib provided a considerable PFS benefit for patients with a deleterious germline breast cancer susceptibility gene (BRCA)1 or BRCA2 mutation [9]. Therefore, Bev, an anti-angiogenic drug, plays an important role in the current and future treatment of ovarian carcinoma.

Anti-angiogenic drugs and chemotherapy drugs with anti-angiogenic effects have been reported to influence vessel remodeling according to different doses and durations of administration [10–12]. Until now, several reports have examined whether paclitaxel with anti-angiogenesis had antitumor effect depending on the differences of the administration [13–15]. However, no studies have examined the association between the schedule of Bev administration and the efficacy of the drug in patients with ovarian carcinoma. Therefore, the aim of this study was to investigate whether the schedule of Bev administration is related to its efficacy by comparing a monthly high-dose schedule and a weekly low-dose schedule.

## Methods

This study was the retrospective analysis. Patients with ovarian carcinoma who received chemotherapy at Ohki Memorial Kikuchi Cancer Clinic for Women, and those who underwent surgery and received chemotherapy at the National Defense Medical College Hospital between 1999 and 2020 were identified. Among them, patients with platinum-resistant recurrence who received a Bev-containing regimen were included in our study. Patients with a history of prior treatment with Bev, those with other carcinomas, and those receiving monotherapy with Bev were excluded.

In this study, the administration method of Bev were 2 patterns. At Ohki Memorial Kikuchi Cancer Clinic for Women, all patients received 100 mg Bev on days 1 and 8 and 200 mg Bev on day 15 monthly. No patients at this hospital received other than this method. They were defined as Group A. On the other hand, at the National Defense Medical College Hospital, all patients received 15 mg/kg Bev administered on day 1 monthly according to our previous report [16]. Patients at this hospital did not undergo other than this method. They were defined as Group B. The administration methods of Bev completely depended on the hospitals. The drugs co-administered with Bev at the two hospitals are listed in Table 1. The standard of the administration was set to the condition that met granulocyte count below grade 1, platelet count below grade 2, hemoglobin level > 7 g/dL, and nonhematological toxicity below grade 1. If patients did not meet the standard on day 1 of the next cycle or on days 7 and 14, the administration on day 1 was postponed by one week and the administrations on days 7 and 14 was skipped. Patients underwent these treatments for a maximum of six cycles unless unacceptable toxicities developed or diseases progressed. The clinical information of the patients was obtained from medical records. Staging was reevaluated using the 2014 International Federation of Gynecology and Obstetrics (FIGO) criteria [17]. Information on residual tumors was obtained from the operation records of the primary surgery. Response Evaluation Criteria in Solid Tumors version 1.1 was used to evaluate the efficacy of chemotherapy [18]. The state of the tumor was evaluated every 8–12 weeks or when symptoms which was suspicious of diseases progression developed. Best response was defined as the most excellent response during treatment. The response rate and the clinical benefit rate was defined as the ratio of complete remission (CR) and partial remission (PR) and the ratio of CR, PR, and stable disease, respectively. Stable duration was defined as the time from the first achievement of the best response to the time of PD. Adverse effects were evaluated using the Common Terminology Criteria for Adverse Events version 4.0 [19]. PFS was defined as the period from the day of Bev administration to the day of death or disease recurrence/progression. OS was defined as the period from the day of Bev administration to the day of death or the last confirmation of survival.

**Table 1:** Details of drugs combined with bevacizumab

Combination drugs	Number of patients	Regimens
Group A	*n* = 44	
Gemcitabine and oxaliplatin	24	400 mg/m^2^ gemcitabine and 40 mg/m^2^ oxaliplatin on days 1, 8, and 15
Pegylated liposomal doxorubicin	10	10 mg/m^2^ pegylated liposomal doxorubicin on days 1, 8, and 15
Eribulin and oxaliplatin	5	1 mg/m^2^ eribulin and 40 mg/m^2^ oxaliplatin on days 1, 8, and 15
Nivolumab	1	100 mg nivolumab on days 1 and 15, every 4 weeks
Paclitaxel and carboplatin	1	175 mg/m^2^ paclitaxel and AUC 5 carboplatin on day 1
Paclitaxel	1	80 mg/m^2^ paclitaxel on days 1, 8, and 15
Trabectedin and oxaliplatin	1	0.25 mg/m^2^ trabectedin and 40 mg/m^2^ oxaliplatin on days 1, 8, and 15
Trabectedin and pegylated liposomal doxorubicin	1	10 mg/m^2^ pegylated liposomal doxorubicin and 0.25 mg/m^2^ trabectedin on days 1, 8, and 15
Group B	*n* = 33	
Gemcitabine	32	1000 mg/m^2^ gemcitabine on days 1, 8, and 15
Paclitaxel	1	80 mg/m^2^ paclitaxel on days 1, 8, and 15

Group A was defined as patients treated with weekly low-dose administration of bevacizumab

Group B was defined as patients treated with monthly high-dose administration of bevacizumab

*AUC* Area under the curve

Statistical analysis was performed using JMP Pro 14 software (SAS Institute Inc., Cary, NC, USA). The chi-square test, Fisher's exact test, and Mann–Whitney U test were used to evaluate the clinical significance of clinicopathological factors. The frequency of adverse effects was compared between patients with grades 0, 1, and 2 and those with grades 3 and 4. PFS and OS curves were generated using the Kaplan–Meier method. Comparison of PFS, OS, and stable duration was performed using the log-rank test. Cox proportional hazards analysis was used for multivariate analysis of PFS. Statistical significance was defined as a *p*-value of < 0.05.

## Results

**Fig. 1:**
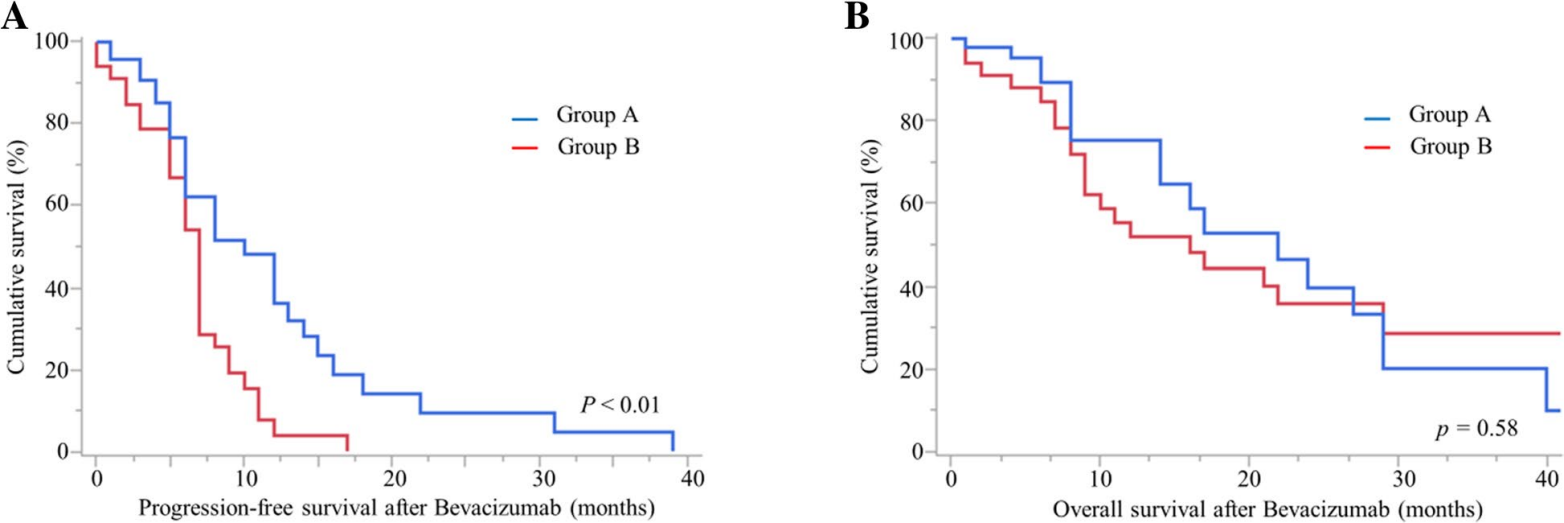
Progression-free survival (**A**) and overall survival (**B**). Group **A** was defined as patients treated with weekly low-dose administration of bevacizumab. Group **B** was defined as patients treated with monthly high-dose administration of bevacizumab

Of the patients, 44 were in group A and 33 were in group B. More patients in group A had an advanced FIGO stage of the disease (*p* = 0.03), and a lower dose of Bev at the first time during the first cycle administration (*p* < 0.01) than in group B (Table 2). The best response, response rate, and clinical benefit rate were not statistically different, but the stable duration was longer in group A than in group B (8 vs. 5 months, *p* < 0.01). The number of patients censored for OS was 26 in group A and 2 in group B (*p* < 0.01). The PFS of group A was better than that of group B (Fig. 1 A, *p* < 0.01); however, the same was not true for OS (Fig. 1B, *p* = 0.58). The number of patients censored for OS owing to treatment, follow-up, and dropout was 26/44 (59%) in group A and 2/33 (6%) in group B (*p* < 0.01). In the multivariate analysis for PFS, weekly low-dose administration of Bev was identified as an independent prognostic factor (Table 3; hazard ratio 0.53, *p* = 0.03).

**Table 2:** Characteristics of patients with ovarian carcinoma according to bevacizumab administration

Characteristics	Group A		Group B		
	*n* = 44		*n* = 33		*p*-Value
Age (years)					0.07
Median (range)	54	(30–77)	61	(29–77)	
FIGO stage (%)					0.03
I	2	(4.5)	7	(21.2)	
II	4	(9.1)	3	(9.1)	
III	33	(75.0)	15	(45.5)	
IV	5	(11.4)	8	(24.2)	
Histological types (%)					0.24
Serous carcinoma	26	(59.1)	18	(54.6)	
Clear cell carcinoma	5	(11.4)	9	(27.3)	
Endometrioid carcinoma	6	(13.6)	1	(3.0)	
Carcinosarcoma	1	(2.3)	1	(3.0)	
Squamous cell carcinoma	1	(2.3)	0	(0)	
Mucinous carcinoma	0	(0)	1	(3.0)	
Adenocarcinoma not otherwise specified	5	(11.4)	3	(9.1)	
Residual tumor (≥ 1 cm) at primary surgery (%)					0.08
Yes	22	(50.0)	10	(30.3)	
No	22	(50.0)	23	(69.7)	
Prior chemotherapy regimens before bevacizumab-containing regimens (times)	3.8	(1–11)	3.5	(1–11)	0.61
Total dose of bevacizumab set for the first time during the first cycle (mg)	400.0	(400)	745.6	(520–1113)	< 0.01
Best response					0.13
Complete remission	3	(6.8)	0	(0)	
Partial remission	6	(13.6)	6	(18.2)	
Stable disease	27	(61.4)	15	(45.5)	
Progressive disease	8	(18.2)	12	(36.4)	
Response rate (%)	20		18.2		0.52
Clinical benefit rate (%)	82		63.6		0.07
Stable duration (months)	8	(0–37)	5	(0–10)	< 0.01

Group A was defined as patients treated with weekly low-dose administration of bevacizumab

Group B was defined as patients treated with monthly high-dose administration of bevacizumab

*FIGO* International Federation of Gynecology and Obstetrics

**Table 3:** Univariate and multivariate analyses for progression-free survival after bevacizumab administration in all cases included in this study

		Univariate analysis		Multivariate analysis	
Variables		Hazard ratio (95% CI)	*p*-Value	Hazard ratio (95% CI)	*p*-Value
Method of bevacizumab administration	Group A vs. group B	0.47 (0.27–0.80)	< 0.01	0.53 (0.29–0.95)	0.03
Age (years)	≥ 50 vs. < 50	1.83 (1.01–3.50)	0.045	1.40 (0.73–2.81)	0.31
Drug combined with bevacizumab	Gemcitabine vs. other regimens	1.70 (0.95–3.25)	0.08		
Histology	Serous carcinoma vs. other histology	0.73 (0.43–1.24)	0.24		
Residual tumor at primary surgery	< 1 cm vs. ≥ 1 cm	0.94 (0.55–1.58)	0.82		
FIGO stage	I, II vs. III, IV	0.98 (0.54–1.90)	0.95		

Group A was defined as patients treated with weekly low-dose administration of bevacizumab

Group B was defined as patients treated with monthly high-dose administration of bevacizumab

*CI* confidence interval, *FIGO* International Federation of Gynecology and Obstetrics

There were fewer patients with grade 3/4 anemia (*p* < 0.01), grade 3/4 neutropenia (*p* < 0.01), grade 3/4 hypertension (*p* < 0.01), intestinal obstruction (*p* < 0.01), and thromboembolic events (*p* < 0.01) in group A than in group B (Table 4). In group A, there were no statistical differences in PFS (6, 12, and 5 months, respectively; *p* < 0.12), response rate (20%, 30%, and 10%, respectively; *p* = 0.55), clinical benefit rate (75%, 100%, and 80%, respectively; *p* = 0.32), and stable duration (4, 9.5, and 3 months, respectively; *p* < 0.13) among patients treated with gemcitabine and oxaliplatin, pegylated liposomal doxorubicin, and other regimens.

**Table 4:** Comparison of adverse effects between the weekly bevacizumab group and the monthly bevacizumab group

Adverse effects	Group A (*n* = 44)		Group B (*n* = 33)		*p-*Value
	Grade 1/2	Grade 3/4	Grade 1/2	Grade 3/4	
Anemia	41	2	20	12	< 0.01
Neutropenia	22	10	12	18	< 0.01
Thrombocytopenia	29	2	14	1	0.99
Febrile neutropenia	0	0	0	0	0.99
aspartate aminotransferase level increased	0	1	4	1	0.99
alanine aminotransferase level increased	2	0	2	0	0.99
Fatigue	37	0	11	0	0.99
Stomatitis	11	0	0	0	0.99
Nausea	32	1	10	2	0.57
Hypertension	5	1	4	11	< 0.01
Thromboembolic event	1	0	2	6	< 0.01
Proteinuria	3	0	9	1	0.43
Gastrointestinal perforation	0	0	0	2	0.57
Intestinal obstruction	0	0	4	6	< 0.01
Cardiac disorder	0	0	1	0	0.99
Hand and foot syndrome	10	0	0	0	0.99
Constipation	15	0	2	0	0.99
Diarrhea	3	0	1	1	0.43
Taste disorder	3	0	1	0	0.99

Group A was defined as patients treated with weekly low-dose administration of bevacizumab

Group B was defined as patients treated with monthly high-dose administration of bevacizumab

## Discussion

The administration method of Bev was different among four clinical trials (GOG-218, ICON-7, OCEANS, and AURELIA) [4/7]. The method of Bev administration was 15 mg/kg body weight triweekly in GOG-218, 7.5 mg/kg triweekly in ICON-7, 15 mg/kg triweekly in OCEANS, and 10 mg/kg biweekly or 15 mg/kg triweekly in AURELIA. The duration of administration was similar across all clinical trials, except AURELIA. The AURELIA trial was the only trial that included patients who received different doses of Bev at different durations; however, the effects of these differences were unclear. Interestingly, although the dose of Bev in ICON-7 was half that in GOG-218, the ICON-7 trial demonstrated a long PFS, similar to GOG-218. Although we could not directly compare ICON-7 and GOG-218, we considered that there was still room for improvement in setting the dose of Bev. In fact, in our study, the group that received weekly low-dose Bev administration had a longer PFS. A low dose of Bev may be effective in patients with ovarian carcinoma.

The efficacy of angiogenesis inhibitors depends on the administration methods and dose in the preclinical model. Continuous treatment can normalize the tumor vasculature and transiently increase the drug uptake of the tumor. Conversely, intermittent treatment might allow the recovery of tumor vascularity between each cycle of drug administration and might facilitate tumor cell recovery from cytotoxic drug treatment [10]. Moreover, lower doses of angiogenesis inhibitors might improve perfusion and drug delivery better than higher doses [11]. In fact, a randomized phase II trial of treatment for metastatic breast carcinoma demonstrated no survival benefit, and differences in response rates were observed among patients who received Bev every week (10 mg/kg), every 2 weeks (10 mg/kg), and every 3 weeks (15 mg/kg) as the co-administered drug of nanoparticle albumin-bound paclitaxel [20]. Meanwhile, a randomized phase II trial of treatment for metastatic colon carcinoma demonstrated that patients who received a lower dose of Bev (5 mg/kg every 2 weeks) had a survival benefit over those who received a higher dose of Bev (10 mg/kg every 2 weeks) as the co-administered drug of fluorouracil and leucovorin [21]. Our study showed that weekly administration of a lower dose of Bev might be effective for platinum-resistant recurrent ovarian carcinoma, as observed for colon carcinoma. The efficacy of the administration methods of Bev might differ among several carcinomas.

In our study, most patients in the monthly high-dose administration group received gemcitabine as the co-administered drug with Bev, whereas patients in the weekly low-dose Bev group received various co-administered agents, which could have affected our results. In a phase III trial examining the efficacy of gemcitabine monotherapy in patients with platinum-resistant recurrence, the overall response rate was 6.1% and the median PFS was 3.6 months [22]. In a multicenter phase II clinical trial investigating the combination therapy of gemcitabine and oxaliplatin in patients with platinum-resistant recurrent ovarian cancer, the overall response rate was 37% and the median PFS was 6.7 months [23]. In a phase III trial determining the effect of pegylated liposomal doxorubicin in patients with platinum-refractory and platinum-resistant ovarian cancer, the overall response rate was 17.4% and the median PFS was 4.0 months [24]. Our study confirmed that there were no statistical differences in efficacy in the group treated with a weekly low-dose administration of Bev. Furthermore, survival analysis demonstrated that the co-administered drugs were not prognostic factors. Thus, the results of our study might be greatly affected by the weekly low doses of Bev, but not by the co-administered drugs.

Several clinical trials have reported that hypertension, thromboembolic events, and gastrointestinal perforation are well-known adverse events associated with Bev [4–7]. In our study, the incidences of these adverse events were lower in the group treated with weekly low-dose administration of Bev, in addition to a lower incidence of hematological toxicities. Therefore, weekly low-dose administration of Bev might decrease the frequency of adverse effects while increasing the anti-tumor effect.

Anti-angiogenic drugs had a synergic effect with poly (ADP-ribose) polymerase inhibitors in the PAOLA-1 trial [9]. In addition, the combination of immune-checkpoint inhibitors and Bev was considered to be promising because VEGF-mediated immunosuppressive effects enhanced immune-mediated antitumor activity [25]. In fact, phase Ib and II trials showed promising effects and tolerable adverse effects. Therefore, anti-angiogenic drugs such as Bev play a central role in chemotherapy for ovarian carcinoma [26, 27]. Future studies should investigate the method of administration of anti-angiogenic drugs.

Our study had several limitations, including its retrospective nature and small sample size. In particular, we could not perform a comfortable analysis of OS. However, our study did not have selection bias in terms of the two administration methods of Bev. Furthermore, weekly low-dose administration of Bev was found to improve PFS and decrease the adverse effects associated with this drug. Thus, we believe that this information needs to be immediately shared and a future study examining the administration methods of Bev is warranted.

## Conclusions

In conclusion, weekly low-dose administration of Bev might be the potential treatment for patients with platinum-resistant ovarian carcinoma in terms of survival benefit and adverse effects. It might be worth performing further studies to examine the methods of administration of anti-angiogenic drugs.

## Data Availability

The corresponding author send dataset according to reasonable request.

## Abbreviations

AURELIA: A Study of Bevacizumab Added to Chemotherapy in Patients With Platinum-resistant Ovarian Cancer
BRCA: Breast cancer susceptibility gene
Bev: Bevacizumab
FIGO: International Federation of Gynecology and Obstetrics
GOG: Gynecologic Oncology Group
PFS: Progression-free survival
ICON: International Collaboration on Ovarian Neoplasms
OCEANS: The Ovarian Cancer Study Comparing Efficacy and Safety of Chemotherapy and Anti-angiogenic Therapy in Platinum-Sensitive Recurrent Disease
OS: Overall survival
PAOLA: Randomized, Double-Blind Controll Trial of Olaparib vs. Placebo in Patients with Advanced Ovarian Cancer
VEGF: Humanized vascular endothelial factor
